# Stability of the volume of air trapped on the abdomen of the water spider *Argyroneta aquatica*

**DOI:** 10.1186/2193-1801-2-694

**Published:** 2013-12-28

**Authors:** Dietrich Neumann, Dietrich Woermann

**Affiliations:** Cologne Biocentre, University of Cologne, Köln, Germany; Institute of Physical Chemistry, University of Cologne, Köln, Germany

**Keywords:** A*rgyroneta aquatica*, Plastron, Physical stability

## Abstract

The water spider *Argyroneta aquatica* lives under water, diving to various depths from time to time. At rest, it breathes air trapped within its diving bell with a hydrophilic surface. Outside their diving bell water spiders trap air on their abdomen under a layer of hydrophobic hair. Is the structure of the layer of hair trapping a volume of air on the abdomen of the water spider *Argyroneta aquatica* under water related to its observed diving depth (of the order of decimetre)? A positive answer to this question is given, based on the law of Laplace in combination with information obtained from SEM- photographs of the abdomen of a water spider.

## Background

The adaptation of the water spider *Argyroneta* to its life under water has been the subject of several recent studies (Neumann and Kureck 2013; Seymour and Hetz 2011; Matthews and Seymour 2010; Schütz and Taborsky 2003). Each of these reports contains a section reviewing the biology of the spider. The adaptation of respiratory systems of spiders is reviewed by Levi (1967). It is the focus of the present study to contribute an answer to the question: Is the structure of the layer of hair trapping a volume of air on the abdomen of the water spider *Argyroneta aquatica* under water related to its observed diving depth?

## Results and discussion

**Figure 1:**
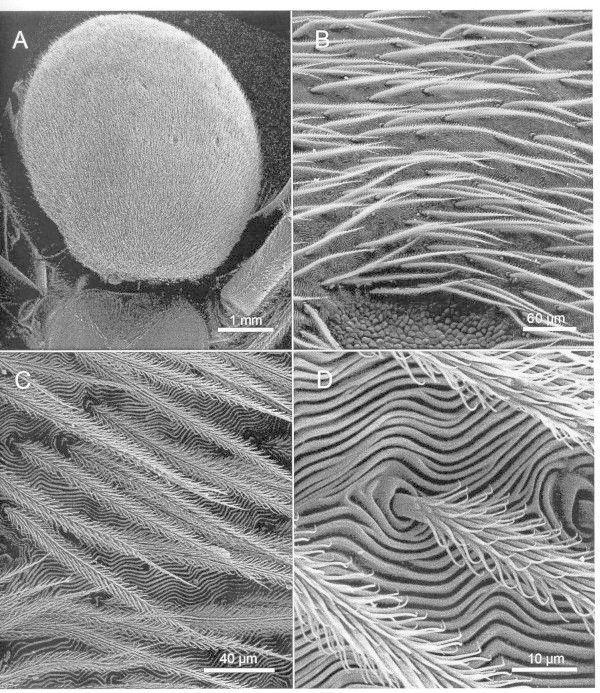
***Argyroneta aquatica*** **, SEM-photos of a full-grown female. A**. Dorsal view of the tightly haired abdomen (syn. opisthosoma) in contrast to the hairless cephalothorax (below only its posterior part with the insertion near the hind leg is shown). The little spots among the hairs on the abdomen represent glandular fields. - **B**. Detailed view of the dorsal abdomen covered with posteriorly inclined feathered hairs above a rippled cuticle, and with rounded area of glandular cells at the bottom of the photo. - **C**. Enlarged view on the field of adjacent feathered hairs. - **D**. Base of feathered hairs (with their barbules) arising from a socket in between the rippled cuticle.

Figure 1 shows scanning electron microscopic (SEM) pictures of the abdomen of *Argyroneta*. They were obtained after gold plating in vacuum using a Hitachi S 520 instrument. The abdomen of the spider is completely surrounded by a loosely packed layer of feathered hair without a large opening. The photographs show that the schematic drawings of the surface of the abdomen of *Argyroneta* found in the literature are simplifications of the actual structure (e.g. Braun 1931; Crome 1951). Kullmann and Stern (1975) have also published SEM pictures of parts of the abdomen of *Argyroneta*.

### Analysis of the stability of the volume of air trapped on the abdomen of *Argyroneta*

We consider a spider which carries under water on its abdomen a volume of air under a layer of hair. The spider is located at a certain distance below the atmospheric air/water surface in its physical equilibrium state (marked by subscript *e*). This is the state into which the spider returns spontaneously after this state had been disturbed by a small external force (e.g. by a force generated by convection of water surrounding the spider or by the movement of the body of the spider). The analysis of the physical stability of the volume of air trapped on the abdomen of the spider is based on a model first proposed by Crisp and Thorpe (1948). It uses the law of Laplace (see e.g. Erbil 2006) in 1-dimensional form. Flynn and Bush (2008) used the 2-dimensional Young-Laplace equation to treat the mechanics of plastron respiration in general terms.

In the present study the law of Laplace is used in the form given by equations (1).1

**Figure 2:**
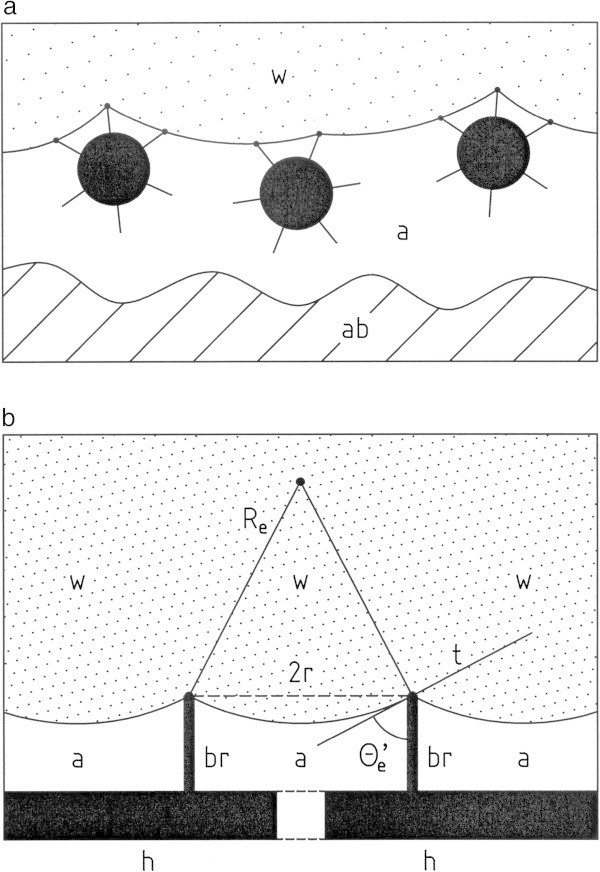
**Structure of the layer of hair covering the abdomen of the spider** ***Argyroneta aquatica*** **(schematic drawing). a**: Vertical cut across three horizontally oriented neighbouring hairs on the abdomen (ab) of the spider. The hairs are part of a layer of the feathered hair with a hydrophobic surface trapping under water (w) a volume of air (a) on the surface of the abdomen. The feathered structure of the hair is presented in Figure 2 **b** by short “bristles” (short straight lines originating from the surface of the long hair covering the abdomen). To be specific, it is assumed that the solid structures have a hydrophobic surface. The tips of the bristles are in contact with water. The locations at which three phases (air - water - bristle) are in contact are marked by black dots (i.e. points of three-phase-contact). The air/water surface connecting two points of three-phase-contact is curved. The origin of the radius of curvature is located within the aqueous phase. **b**: Side view on the air (a)/water (w) surface of two short sections of the same or of two different long hairs (h) with a hydrophobic surface. Each section of the surface carries a bristle (br). Two points of three-.phase-contact are marked by small black dots which are a distance (2 *r*) apart. *R*_*e*_ is the radius of curvature of the air/water surface connecting two points of three-phase-contact. The straight line marked by the symbol *t* is the tangent to the curved air/water surface at one of the points of three-phase-contact. The contact angle θ_e_ is measured across the liquid phase (by definition). The drawings refer to an arbitrary value of θ_e_ > 90°. The contact angle θ_e_ *'* is given by: θ_e_ *'* = 180° − θ_e_.

The schematic drawings shown in Figure 2 relate the parameter θ_e_, *r* and *R*_*e*_ in equations (1) to the proposed model of the structure of the layer of feathered hair covering the abdomen of the spider. The equations (1) are written in a form to be applicable to a solid structure with a hydrophilic surface as well as to a structure with a hydrophobic surface. θ_e_ is the contact angle air-water-solid: hydrophilic surface, θ_e_ < 90°; hydrophobic surface, θ_e_ > 90°. *P*^*w*^ is the pressure within the water (*w*) at the location of the spider. *P*^*a*^ is the corresponding pressure of the air (*a*) trapped on the abdomen of the spider. γ_a/w_ is the air/water surface tension (γ_a/w_ = 72.5 ⋅10^−7^ J cm^−2^ at 25°C). It is a positive quantity for physical reasons. The object *r* represents a length characterizing the structure of the layer of hair on the abdomen of the spider (see Figure 2b). ρ_w_ is the density of water (ρ_w_ ≈1 g cm^-3.^). *g* is the gravitational constant (g = 9,81 m s^−2^). The object *x* (≥ 0) is a space coordinate running perpendicular to the external atmospheric air/water surface (*x* = 0). The object x_*e*_ is the safe diving depth defined by equations (1). Based on equations (1) the dependence of *x*_e_ on the structure parameter *r* can be written in a compact form:

x_e_ = C/*r* with C = − (2 ⋅γ_a/w_⋅cos (θ_e_))/(ρ_w⋅_⋅*g*). If the surface of the hair is hydrophobic (θ_e_ > 90° (i.e. cos (θ_e_) < 0), the safe diving depth x_e_ increases with decreasing values of the structure parameter *r*. For *x* > *x*_e,_ equations (1) are violated: The air trapped below the layer of hair on the abdomen of the spider is no longer in a physical stable state. The trapped air could be replaced by water.

The following conclusions are drawn from equations (1):

1. A volume of air trapped on the abdomen of the water spider is in a stable (equilibrium) state if the three terms in equations (1): *P*^*w*^, [*P*^*w*^ −*P*^*a*^]_e_ and {− 2·γ_a/w_ ·_·_cos (θ_e_)/*r*} have the same (positive) value. The term {− 2·γ_a/w_·_·_cos (θ_e_)/*r*} is positive, if the contact angle θ_e_ at the points of three-phase-contact (air-water-hair) is larger than zero: (i.e. θ_e_ > 90°, cos (θ_e_) < 0). The surface of the hair is hydrophobic. Consequently the pressure difference [*P*^*w*^ −*P*^*a*^]_e_ has a positive value. The hydrostatic pressure *P*^w^ at a point below the atmospheric air/water surface is positive. The length *r* is a positive quantity by definition. The surface of the hair on the abdomen of the spider must be hydrophobic to trap a volume of air on the abdomen in a stable state. This finding is in agreement with common knowledge (e.g. Crome 1951; Wesenberg-Lund 1939).
  In contrast the surface of the wall of the diving bell of *Argyroneta* must be hydrophilic to trap air within the diving bell in a stable state. This is the consequence of the fact that the diving bell must have a large opening at its bottom to allow the spider to enter or leave its diving bell (Woermann 2010).
2. The entire curved air/water surface covering the abdomen of the water spider is supported by the tip of feathered hair with a hydrophobic surface enclosing the entire abdomen of the spider without a large opening (i.e. an opening with a diameter of the order of 1 mm or larger).

### Estimated values of the safe diving depth x_e_ using equations (1)

**Figure 3:**
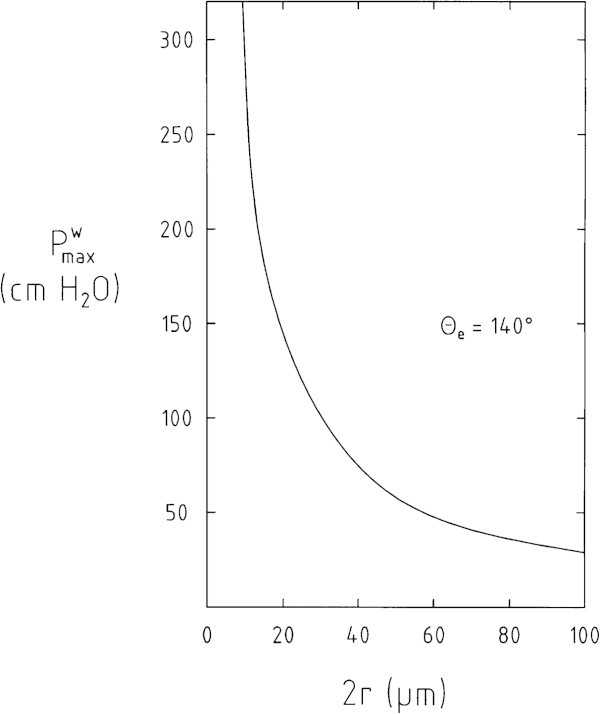
**Plot of the hydrostatic pressure** ***P*** ^***w***^ **up to which air is trapped under a layer of hydrophobic hair on the abdomen of the spider in a stable state as function of the parameter (2** ***r*** **) based on equations (**1**).** Contact angle θ_e_ = 140°. *P* ^*w*^ = *ρ*_w_ ⋅*g* ⋅ *x*_e_) expressed in units of the length (cm) of a vertically oriented water column).

Figure 3 shows a plot of the hydrostatic pressure *P*^*w*^ = (ρ_w_ ⋅*g*⋅ *x*_e_) as function of the parameter (*2 r*) in the range 5 μm < (< 2 *r*) < 100 μm (see Figure 1) with θ_e_ = 140°, based on equation (1). The contact angle θ_e_ could not be determined accurately by independent experiments. But it is observed that θ_e_ is well above θ_e_ > 90°: Droplets of water positioned on the dry surface of the abdomen of a spider rolled off without leaving a trace of water on the abdomen. It is assumed that θ_e_ has a value in the range of 120° ≤ θ_e_ ≤ 160°. The range of relevant structure parameter (2 *r*) used for the estimation is taken from the electron microscopic pictures shown in Figure 1: 5 μm < (2 *r*) < 100 μm.

Which information is given by the curve shown in Figure 3 with θ_e_ = 140°?

For example: Choose on the abscissa of Figure 3 the point (2 *r*) = 40 μm. The corresponding ordinate is *P*^w^ = 0.75 m. This is the pressure exerted by a vertically oriented water column of a length of *x*_e_ = 0.75 m. It is the value of the “safe” diving depth of the spider given by the proposed model. This implies that there is no location on air/water surface on the abdomen of the spider where the distance (2 *r*) between the tip of two feathered hairs is lager than 40 μm (i,e. (*2r*) ≤ 40 μm). - The data compiled in Table 1 show that the dependence of the pressure *P*^*w*^ (= ρ_w_ ⋅*g* ⋅ x_e_) on the contact angle θ_e_ is only weak.

**Table 1:** **Compilation of estimated values of the hydrostatic pressure** ***Pw*** **based on equations (** **1** **)**

2 ***r*** [μm]	θ_e_= 130^ο^	θ_e_= 160^ο^
	[cm water column]	[cm water column]
5	372	545
10	186	272
20	193	136
50	37	54
80	23	34
100	19	22

The hydrostatic pressure *P* ^*w*^ is expressed in units of the corresponding length [cm] of a water column. θ_e_: contact angle. 2 *r*, parameter characterising the structure of the layer of hydrophobic hair covering the abdomen of the spider.

There are only few reports in the literature referring to depths at which *Argyroneta* has been observed under water: *Argyroneta* has been collected from a water body with a depth of 0.5 m to 1.0 m (Schütz and Taborsky 2003); Wesenberg-Lund (1939) reports that nets produced by this water spider have been found several decimetre below the atmospheric air/water surface. The water spiders which have been observed in the present study have been kept (together with shoots of the water plant Elodia) in cylindrical glass containers filled with water to a level of 5 dm. The spiders inhabited the entire available water space.

The proposed model in form of equations (1) is used to estimate for a given value of the contact angle θ_e_ (e.g. θ_e_ = 140°) and a given range of diving depths of the spider (*x*_e_ < 10 dm), the corresponding values of the structure parameter (2*r*): This leads to values of the structure parameter: (2 *r*) ≥ 30 μm). This value is within the range of values of (2 r) taken from the SEM picture of the abdomen of the spider (5 μm < (2 *r*) < 100 μm). This finding gives a physical justification for the intuitive expectation that the observed diving depths of *Argyroneta* are safe diving depths. For diving depths *x*_*e*_ > 10 dm the corresponding range of values of the structure parameter (2 r) is shifted to smaller values: (2r) < 30 μ m. The equations (1) are not suited for an estimation of maximum diving depth of the spider.

### Consequences

**Figure 4:**
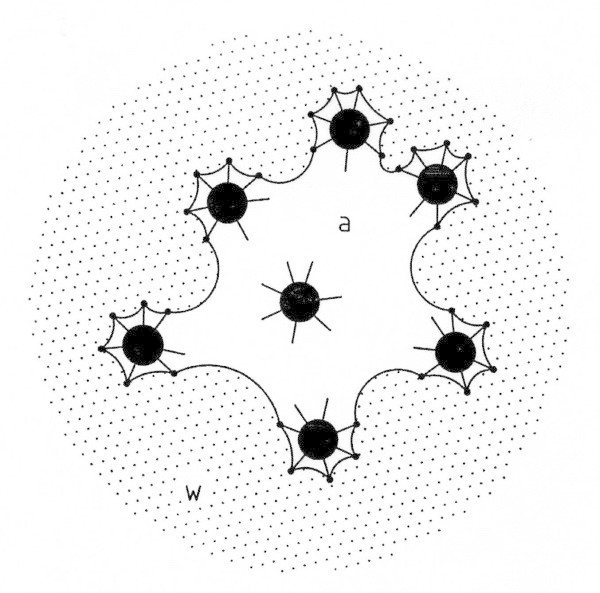
**Replenishment of the volume of air trapped on the abdomen of** ***Argyroneta*** **.** Schematic drawing of a top view onto an atmospheric air (a)/water (w) surface during a replenishment.

The replenishment of the air forming the plastron of the spider is only possible if the surface of hair trapping a volume of air on its abdomen has a hydrophobic surface. Let us assume that a replenishment of trapped air becomes necessary. It is observed that in this case the spider moves in the direction of decreasing diving depth. On its way upwards the pressure *P*^w^ acting on the spider decreases. Consequently the radii *R*_*e*_ of curvature on the air/water surface on the spider’s abdomen increase (see equations (1)). Before the spider reaches the atmospheric air/water surface it turns around (180°) in such a manner that the atmospheric air/water surface is broken by force in such a way that the ends of hairs covering the lower part of the abdomen stick out into the outer atmosphere. The outer atmosphere comes into direct contact with the air remaining in the plastron. A replenishment of the trapped air can take place under a nearly isobaric condition. A schematic drawing of this situation which lasts only for a few seconds is shown as Figure 4.

Furthermore, the adaptation of the spider to its life under water makes it necessary for it to fill a new diving bell with air during its construction and to replenish the air in an already existing diving bell. For these actions it is necessary for the spider to fetch air from the outer atmosphere and to transport it under water to the location where it is needed. These actions are distinctly different from that required to replenish the plastron. It is outside the scope of the present study to analyse these actions in detail. They have been observed and documented repeatedly (e.g. Braun 1931; Crome 1951; Heinzberg 1974; Nuridsany and Pérennou 2004. They have also been observed by DW in the cause this study). There cannot be any doubts that the external, rough, hydrophobic layer of hair on the abdomen of the spider is in contact with the fetched air during its transport by the spider under water in the direction of the force of gravity. During this transport the air is exposed to the force of buoyancy acting in the opposite direction. The volume of air must be stabilised not to get lost. It is known that air adheres to a hydrophobic surface, minimizing the surface energy of the water/hydrophobic surface. In addition the spider uses its 4th pair of hind legs as tongs with a hydrophobic surface. Nevertheless the air is occasionally lost by buoyancy.

### Remarks

1. It is noticed (see Figure 1 and Kullmann and Stern 1975) that the surface of the abdomen of *Argyroneta aquatica* has a corrugated structure. The body of the spider is constantly exposed to a hydrostatic pressure which changes with the diving depth. The corrugation could have the function to increase the stiffness of the surface layer of the abdomen. Without corrugation the deflection of the surface of the abdomen could harm the function of the organs in the spider’s abdomen.
2. Are the observed diving depths of the spider maximum values? In an attempt to contribute to an answer of this question the following two experiments had been carried out at the beginning of the present study: A pressure vessel made of glass was filled with water saturated with air. For each experiment a single spider together with a shoot of a water plant was placed into the pressure vessel. The vessel was closed and connected to a device to keep a given value of a hydrostatic pressure constant in time (in combination with a pressure gauge and a pressurised bottle filled with air). The pressure difference Δ *P* between the interior of the vessel containing the spider and the outer atmosphere was increased slowly step by step from Δ*P* = 0 bar up to Δ *P* = 1.1 bar (and up to ΔP = 1.3 bar, in the second experiment). Each run took about 2 h. The spiders did not move during that period of time. At the end of each run both of the spiders were dead, probably caused by a rupture of an internal organ within their abdomens. These experiments had been broken off. *Argyroneta* belongs to the group of endangered species.- At present, there is no definite answer to the question stated above.
3. Comparison of the property of the structure of the plastron of the water bug *Aphelocheirus* with that of the water spider *Argyroneta*: The form and the mechanical stability of the plastron of the water bug differs from that of the water spider. Part of the structure of the plastron of the water bug is a rigid solid, It has the form of a bowl (turned upside down) which is in contact with the air forming the plastron. The compressibility of air is given by  (ideal gas), (T, temperature, V, volume). The air in the plastron of the water bug is compressible and “not collapsible”. It can be expected that water bugs can be observed at depths in the range of meters.
  This is in agreement with reported observations (Carbonell et al. 2011).- The plastron of a water spider is formed of “soft” materials. Part of its structure is an air/water surface stabilized by a layer of hair with a hydrophobic surface. It can be expected that the Stiffness of the abdomen of the spider is limited: The plastron of the spider is “collapsible”.
4. The water spider is able to trap air in a stable state within two structures with different surface properties: Within its diving bell with a hydrophilic wall and on its abdomen below a layer of hair with a hydrophobic surface. These abilities in combination are part of the adaptation of the spider to its life under water. They depend on each other and might have evolved in parallel.
5. The preferential adsorption of surfactant molecules onto a hydrophobic solid surface changes the surface property of the solid from hydrophobic to hydrophilic. This process takes place in the habitat of a spider if the water is polluted by surfactants. This change of surface property leads to suffocation of the spider.

## Methods

The methods which enable a replication of this study are given in the first sentences in the section “Results and discussion;” of this contribution and in Neumann and Kureck 2013.
